# Maintaining physical activity in people with long-term conditions following engagement in physical activity referral schemes: barriers, enablers, and intervention strategies

**DOI:** 10.1186/s12966-025-01802-y

**Published:** 2025-07-23

**Authors:** James P. Gavin, Luisa C. Holt, Paul E. Muckelt, Euan Sadler, Suzanne McDonough, Mary Barker

**Affiliations:** 1 School of Health Sciences, University of Southampton, Southampton, UK; 2 RCSI University of Medicine and Health Sciences, Dublin, Ireland; 3 Faculty of Medicine, University of Southampton, Southampton, UK

## Abstract

**Background:**

There is currently limited evidence on the long-term effectiveness of referral programs from healthcare to community sectors to help people with long-term conditions (LTCs) maintain physical activity. This study aimed to (i) identify the support needs of people with LTCs in maintaining physical activity following physical activity referral schemes (PARS); (ii) understand healthcare professionals’ experiences of referring and delivering on PARS, and; (iii) begin co-designing a pathway integrating healthcare, leisure and community to maintain physical activity for LTCs.

**Methods:**

A qualitative design involved (i) stakeholder mapping, ii) interviews with people with LTCs (*n* = 15) and healthcare professionals (*n* = 9), and; (iii) workshops with participants with LTCs (*n* = 6) and multi-sectoral professionals (*n* = 15). Workshops involved multi-sectoral professionals responsible for designing, delivering, referring and/or commissioning PARS across health, leisure and community sectors. Interviews and workshops were thematically analyzed, with themes mapped to the Capability, Opportunity, Motivation–Behavior (COM-B) model.

**Results:**

Participants with LTCs recognized that although PARS increased their psychological and physical capabilities, aging and symptoms impacted their day-to-day capabilities and motivation. Professional feedback and monitoring, complemented by peer support, were essential in mitigating this and maintaining physical activity behaviors. Healthcare professionals were challenged by the time taken to identify community-based opportunities, navigating referrals, and lacked sufficient feedback on client progress, but felt group activities, enjoyment and social support were integral. Workshops highlighted the need for an integrated pathway from medical intervention to community-based support, to improve physical activity maintenance for LTCs. Better partnership working between multi-sectoral agencies was prioritized to increase scheme accessibility, including simple, standardized referrals. Using behavior change techniques to personalize support was also valued, including ‘healthy conversations’ training for healthcare professionals.

**Conclusions:**

Healthcare, leisure, and community sectors should collaborate to create accessible local activities that promote social support, feedback and monitoring, and public partnership. Healthcare professionals should consider individual capabilities, foster motivation during and after PARS, and facilitate networks for resources and knowledge sharing.

**Supplementary Information:**

The online version contains supplementary material available at 10.1186/s12966-025-01802-y.

## Background

In England, over 15 million people (> 25% of the population) live with an incurable long-term health condition (LTC) [1], which requires management with medication or other therapies [2]. Physical activity is fundamental in the management of LTCs [3]. Increasing activity levels can help maintain strength and balance limiting falls risk, reduce anxiety and depression [3], and help manage pain [4–6]. Long-term maintenance of physical activity is also crucial for disease prevention [7, 8], but in the United Kingdom (UK), the prevalence of multimorbidity, defined as two or more LTCs, is increasing [8], and patients with multiple conditions are more likely to be physically inactive [9]. It is, therefore, important to move beyond a single-condition approach to support people with LTCs to engage in long-term activity.

The short-term efficacy of physical activity programs encompassing healthcare, voluntary and community sectors is well-documented [10–12], however the long-term (> 3 months) benefits remain unclear [10, 13, 14]. This may be because programs focus on initiating physical activity but lack the necessary personalized components to support behavior change for long-term maintenance [15, 16]. To maintain behavior change beyond 12-months, National Institute for Health and Care Excellence (NICE) guidelines [17] recommend that individuals: (i) receive regular feedback and monitoring; (ii) have well-established action plans for relapses; (iii) consider how their physical environment can be adapted; (iv) have social support; and (v) are supported to develop routines promoting new behavior, for at least 12 months. Programs typically rely on in-person follow-ups and professional supervision for maintaining longer-term physical activity post-intervention [18], with digital tools appearing consistently effective, and offering access to social support from peers and professionals.

Programs to support longer-term physical activity adherence have little or no effectiveness, small effect sizes which decline over time, and fail to assess maintenance outcomes [14, 19, 20]. It may be that programs do not provide support for long enough, and therefore fail to support habit formation to promote positive behavior change, nor tailor support to promote autonomy in physical activity. Building autonomy and self-efficacy are important in the transition from behavior initiation to maintenance for people with LTCs. Motivational interviewing, counselling and goal setting can support these [21, 22] in maintaining behavior change [12, 20], as they promote flexibility to tailor physical activity to LTCs allowing choice/volition (autonomy), experience of mastery (competence), and opportunity for social connection (relatedness) which motivate behavior [23].

Given that there is no agreement on what makes for effective physical activity maintenance [21, 24], the COM-B model [25] and the associated Behavior Change Wheel can be used to design and evaluate programs incorporating behavior change, with the aim of improving long-term adherence and physical activity levels [26, 27]. The COM-B model suggests that the likelihood of performing a behavior is influenced by three components: a person’s capability (physical and psychological), opportunity (physical and social) and motivation (automatic/habitual and reflective) [25]. To develop an intervention to facilitate physical activity maintenance, this model can be used to identify deficits in one or more aspects of these components, and the Behavior Change Wheel used to suggest intervention options (including policy domains for delivery). For example, if someone is unable to get themselves to a gym to exercise (physical opportunity), a solution might be to offer transport (environmental restructuring).

The Maintenance Of physical acTivity beHavior (MOTH) program aimed to: (i) identify existing digital tools that support people with LTCs to maintain physical activity [28, 29], (ii) understand the factors facilitating and inhibiting their use in the National Health Service (NHS), and (iii) understand the support needs of participants with LTCs and professionals to maintain physical activity following Physical Activity Referral Schemes (PARS). Overall, the program sought to develop an intervention incorporating digital and non-digital support to help people with LTCs maintaining activity. In this paper we report the ‘non-digital’, PARS component, which are programs that integrate health, social and community care, to support LTC management [10, 12, 30]. The effectiveness of PARS in supporting maintenance of physical activity for LTCs is unclear [22, 31]. This may partly due attributable to the heterogeneity of schemes, in terms of setting, duration and intensity, and scheme components, including whether they contain screening and/or brief advice [22], how they are funded, and in age and socio-economic status of participants [10, 12]. PARS currently run across the UK but fail to tailor programs to support long-term adherence for LTC participants [10].

In the UK, there is growing impetus for the integration of healthcare, social and community services to support habitual physical activity levels, and address inequalities, in local communities [11, 32]. People living with LTCs and those from marginalized groups [11, 33] experience substantial barriers to undertaking prescriptive programs [34] like PARS [30], including comorbidities, transport costs, and caring responsibilities. Little is known about how best to design services to support individuals with LTCs to stay physically active in the long-term and across different care settings [11, 28]. Therefore, this study aimed to:

- Identify what is needed to support people with LTCs to maintain physical activity on completion of a PARS.
- Understand healthcare professionals’ experience of referring to and delivering PARS for people with LTCs, and;
- Begin co-designing the components of an integrated care pathway through healthcare, social and community services to support physical activity maintenance for people with LTCs.

## Methods

### Research design

This exploratory qualitative study was part of the MOTH program which aimed to develop an intervention, incorporating digital tools [28, 29] to support long-term, physical activity maintenance for people living with LTCs (ISRCTN: 16805986I). We defined maintenance of physical activity as lasting ≥ 3 months post-intervention [29]. This paper reports data collected from three linked phases:

(i) Stakeholder mapping - identifying multi-sectorial stakeholders to assist in recruiting healthcare professionals and people with LTCs.
(ii) Interviews - people with LTCs and healthcare professionals to understand the experiences and support needs of undertaking or referring to a PARS, and;
(iii) Workshops - people with LTCs and multi-sectoral professionals to begin co-designing a pathway to support physical activity maintenance for people with LTCs.

The study was approved by the University of Southampton (ERGO ref: 76270.A4) and the UK NHS Health Research Authority ethics committees (IRAS ref: 288651), and conducted in Wessex, UK.

### Phase i: Stakeholder mapping

Initially, the research team drew upon their professional networks and drafted a list of potential multi-sectoral organizations and institutions that involved professionals involved in the design, referral and/or delivery of PARS for people with LTCs. Researchers, LH and PM, then emailed contacts in Clinical Commissioning Groups, the Wessex Clinical Research Network, Solent NHS Trust, clinical academic networks, and health and wellbeing providers in local council, leisure organizations, voluntary community and social care sectors across Wessex, UK, with the recruitment materials. These included explanations of the study aims, what participants would be asked to do, and potential outcomes of the study.

**Fig. 1:**
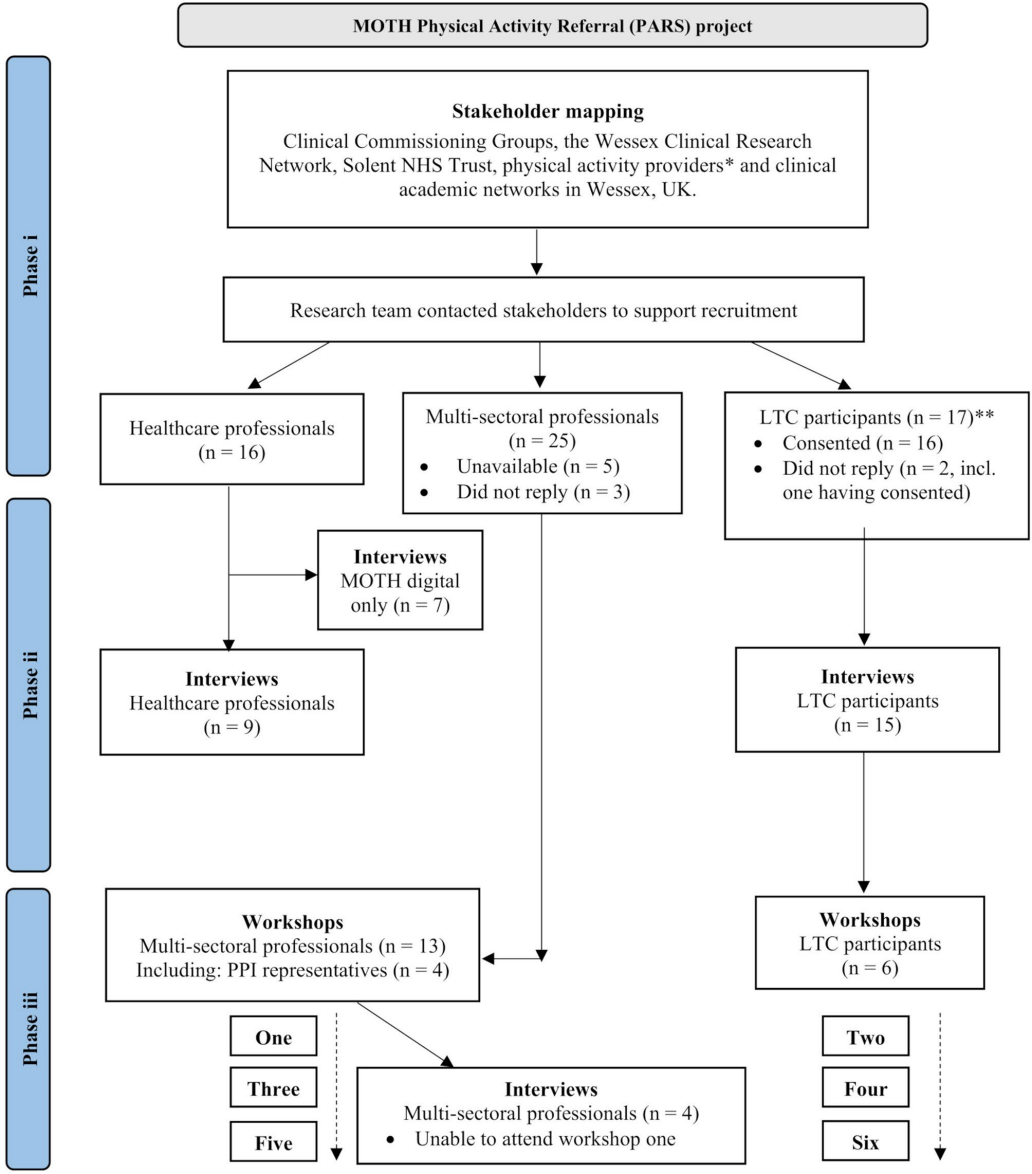
Flow chart – research phases and recruitment *Everyone Active; Places Leisure; BH Live; Active Nation; Eastleigh Borough Council; Active 4 Health (Dorset Council); Basingstoke and Alton Cardiac Rehabilitation Centre **It is not known how many people with LTCs were approached by service providers for phase ii interview recruitment LTC, Long-term condition; MOTH, Maintenance Of physical acTivity beHavior; NHS, National Health Service; PPI, Public and patient involvement; UK, United Kingdom

A total of 16 healthcare professionals, and 25 multi-sectoral professionals, which included seven PARS providers, expressed an interest in the project after being approached by the research team (see Fig. 1). In brief, nine healthcare professionals interviewed as part of the MOTH digital project [28] were asked additional questions about non-digital interventions for physical activity maintenance. Participants with LTCs attending the workshops were a self-selected subset from the phase ii interviews. Multi-sectoral professionals attending the workshops were selected from amongst those who took part in the phase i stakeholder mapping, on the basis of their availability.

### Phase ii: The experiences and support needs of people living with LTCs and healthcare professionals using PARS

#### Design

We conducted one-to-one, semi-structured interviews with healthcare professionals to identify and categorize the needs of those referring to a PARS, and discuss the components/requirements for a follow-on intervention (including features, functions and delivery expectations). Secondly, we interviewed people with LTCs to identify their experiences and support needs following participation in a PARS.

#### Sampling and recruitment

Healthcare professionals were recruited between January-December 2021. Purposive sampling was used for healthcare professionals to reflect diversity in geographical location, demographics and professional experience (i.e., LTC specialism/role) [35, 36].

Participants with LTCs were recruited between October-December 2022, using convenience sampling. Exercise professionals involved in the phase i stakeholder mapping gave out letters of invitation (which included the study email address) to people with LTCs who had participated in a PARS they had delivered. Interested participants returned a reply slip and were then screened for eligibility by a researcher (LH). Eligible participants were contacted individually to gain consent. Eligible participants were to have started a PARS within the last 3–24 months, with diagnosis of one or more of the following LTCs: diabetes, arthritis, stroke, overweight/obesity (30.0–39.9 kg/m^2^), chronic obstructive pulmonary disease (COPD), mental health problems (depression, anxiety, and/or stress), hypertension, osteoporosis, and/or cardiovascular disease according to the UK Exercise Referral Toolkit [37]. We aimed to recruit 20 people with LTCs, based on previous interview studies exploring physical activity behaviors and interventions with this cohort [38, 39].

#### Procedures and data collection

Interviews were conducted by two researchers (PM, LH), using separate interview guides for healthcare professionals (April 2021-January 2022) and people with LTCs (November 2022-January 2023) (Additional file 1). LTC interviews explored three phases of PARS involvement: (i) experiences of referring into or participating in a PARS; (ii) post-PARS experiences and physical activity behavior maintenance; and (iii) barriers and facilitators to long-term engagement in physical activity. Healthcare professional interviews focused on: (i) experiences recommending and/or supporting a physical activity intervention (including PARS); and (ii) barriers and facilitators to engagement in an intervention to support physical activity maintenance for people with LTCs post-PARS. Interviews lasted 45–60 mins and were either held online via Microsoft Teams and audio-recorded using Teams software, or via telephone and recorded using a digital recorder. Socio-demographic measures were age, gender, and location of practice, and/or commissioning area of each participant, for Phase ii interviews and Phase iii workshops. Participants with LTCs completed the Godin Leisure Time Exercise Questionnaire [40] to estimate habitual physical activity levels (Table 1). Written and verbal informed consent were obtained from participants by either PM, LH or PC*.

### Phase iii: Co-design workshops with people living with LTCs and multi-sectoral professionals

#### Design and recruitment

Phase ii participants with LTCs and phase i multi-sectoral professionals were invited to participate in a series of three stakeholder workshops, respectively (six workshops total). Workshops 1 and 2 were run in January 2023, 3 months after completion of Phase ii, and involved feedback on the interview findings, which identified a need for an ‘integrated pathway’ intervention to support physical activity maintenance for people with LTCs. Three months later, workshops 3 and 4 involved discussing the precise requirements for a pathway with participants. Workshops 5 and 6 focused on (i) potential program components to support community-based, physical activity maintenance; and (ii) how to best to evaluate services demonstrating different types of partnership working (see Additional file 2 for workshop schedules). Four professionals who were unable to attend the first workshop subsequently participated in three interviews (with two professionals interviewing together) to ensure their input into the last two workshops.

#### Procedures and data collection

Two pairs of workshops, one for LTC public (either workshop 2, 4 or 6; each 2 h) and one for multi-sectoral professionals (either workshop 1, 3 or 5), were delivered on the same day on three separate occasions. Workshops were facilitated by LH, with MB, ES or JG co-facilitating. LTC public workshops 4 and 6 were held in-person, in response to participant preference after an initial online workshop, audio-recorded and transcribed by an independent provider. Professional workshops were held online via Microsoft Teams, recorded, and automatically transcribed using Teams software. Computer-generated transcriptions were checked, with mis-recordings corrected before analysis and anonymization. Facilitator (LH) took written fieldnotes during workshops, which described the setting, group dynamics, and interactions in the group discussions. Following each workshop, participants were sent the summary report of the discussion for their checking.

#### Data analysis – Phases ii and iii

Interviews and workshop transcripts were thematically analyzed after each period of data collection, using QSR NVivo (Version 10) software. Transcripts were read thoroughly by PM (healthcare professionals, *n* = 9) or LH (healthcare professionals, *n* = 9, people living with LTCs, *n* = 15, six workshops and three related interviews). They coded the transcripts, re-reading them for familiarization, developing initial codes and themes, before using the NVivo code clusters to create a coding map [41, 42]. PM and LH checked their coding together, and with an independent researcher (ES) to resolve any disagreement. Ongoing discussions involving LH, MB and ES organized and synthesized the initial codes into appropriate themes and subthemes. Themes and sub-themes from interviews with participants with long-term conditions and from the workshops were drawn together in a schematic map explaining the relationship between themes and sub-themes in both datasets. Analyses concluded with LH, ES, MB, SMcD and JG reviewing and discussing face validity, and coherence of the interpretation according to how it met the research aims.

This process produced two key themes in the data: (i) factors influencing supported self-management of LTCs; and (ii) factors supporting better partnership working as part of an integrated care pathway. These themes are discussed in detail in the Results section of this paper. Codes from the first of these themes were then classified under the domains of the COM-B model describing key influences on supported self-management of LTCs and physical behavior in particular [25]. Interview codes were therefore classified according to whether they referred to factors that affected capability, opportunity, motivation or behavior. Workshop transcripts were only coded using inductive thematic analysis [42], as their aim was to begin intervention co-design, informed by behavioral findings from phase ii interviews. Classification enabled identification of PARS components and the impacts of participating in, or referring to PARS, that contributed to engaging in/or maintaining physical activity.

## Results

### Participant characteristics

Fifteen people living with LTCs (aged 22–89 years) and nine healthcare professionals (seven general practitioners [GPs], one nurse and one commissioner; aged 33–63 years) were interviewed (Table 1). Over half of participants with LTCs were men, 10 lived with co-morbidities, 12 were retired, 11 described themselves as physically active and all as white British.

Healthcare professionals were predominantly GPs (*n* = 7) and supported people with LTCs to stay physically active by providing written information, advice, and activities (such as exercise instructions). Some referred, others encouraged self-referral, and/or signposted them to other organizations (e.g., Macmillan Cancer Support) for condition-specific support.

Workshops included six adults with LTCs (Table 1), and eight multi-sectoral professionals working in health and social care (*n* = 3), leisure (*n* = 4), voluntary/community sectors (*n* = 1), and patient and public involvement (PPI) representatives with lived experience (*n* = 4). Four multi-sectoral professionals (1 GP, 2 health coaches, 1 leisure) were interviewed separately after workshop one, due to work patterns limiting their ability to participate in the workshops (Table 1).

**Table 1:** Characteristics of phase ii interview participants (those with long-term conditions [LTCs] and healthcare professionals) and of phase iii workshop participants (those with LTCs and multi-sectoral professionals)

Phase ii interviews				Phase iii workshops				
People with LTCs (*n* = 15)*		Healthcare professionals (*n* = 9)		People with LTCs	(*n* = 6)		Multi-sectoral professionals	(*n* = 17)
**Age range (y)**	22–89	**Age range (y)**	33–63	**Gender**	Primary LTC**	**Gender**		**Sector**,** location**
Women	6	Women	5	Woman	Respiratory	Woman		Academia, West Midlands
Men	9	Men	4	Man	Musculoskeletal	Woman		H&SC, Dorset
**Ethnicity**		**Ethnicity**		Man	Neurological	Woman		H&SC and academia, Dorset and Hampshire
White British	15	Not disclosed	-	Man	Musculoskeletal	Woman		Leisure, Dorset
Primary LTC**		**Primary role**		Man	Cardiovascular	Woman		H&SC, Dorset
Musculoskeletal	3	General practitioner (GP)	7	Man	Cardiovascular	Woman†		Leisure, Hampshire
Cardiovascular	4	Nurse	1			Woman		VCSE, Hampshire
Respiratory	3	Commissioner	1			Man		Leisure, Dorset and Hampshire
Neurological	5					Man		PPI representative (lived experience of a LTC)
**Living with co-morbidities**	10	**Location**				Man		PPI representative (lived experience of a LTC)
**Employment status**		Hampshire	4			Woman		PPI representative (lived experience of a LTC)
Employed (full-time)	1	Dorset	3			Woman		PPI representative (lived experience of a LTC)
Employed (part-time)	2	Buckinghamshire	2			Woman		H&SC, Dorset
Retired	12					Woman		Leisure, Hampshire
**Highest level of education**	1					Man†		H&SC - GP, Hampshire
Other professional qualification	5					Woman† & woman†		H&SC – health coaches, Hampshire
University degree	5					†Interviewed separately after workshop 1 (*n* = 4)		
Further secondary (i.e., sixth form/college)	6							
Secondary	3							
**Current domestic status**								
Married/living with partner	11							
Single	2							
Divorced/separated	2							
Current level of physical activity participation***								
Active	11							
Moderately active	4							
Insufficiently active/sedentary	0							

*All (*n* = 15) referred from Hampshire, UK. **Primary LTC for which they were referred to an activity scheme. ***Self-rated using the Godin Leisure-Time Questionnaire [40]. GP, General practitioner; H&SC, health and social care; LTC, long-term condition; PPI rep, patient and public involvement representative; VCSE, voluntary, community and social enterprise

Experiences of PARS broadly related to factors (i) influencing supported physical activity maintenance for LTCs; and (ii) supporting better partnership working, as part of an integrated care pathway. The coding schedule for analysis of interviews using the COM-B model is shown in Additional file 3. Key determinants of behavior for physical activity maintenance based on the COM-B model are summarized below, and presented in Table 2.

**Table 2:** Factors influencing supported physical activity maintenance for individuals with long-term conditions (LTCs) aligned to the COM-B model

Domain*	Theme	Experience
Capability	Physical capability	*Physical levels change over the lifespan whilst living with LTC(s)* *Importance of habitual physical activity* *Relationship between physical and psychological capabilities* *Need for continued personalized support as physical capability changes*
Capability	Psychological capability	*Awareness that physical activity is necessary to limit the impacts of aging and support LTC management.*
Opportunity	Physical opportunities	*Availability of resources* (specifically time, money, equipment) *Accessibility* (i.e., transport and whether services/facilities address their needs)
Opportunity	Social opportunities	*Sociocultural environments* *Group sessions* – motivating and accessible (particularly offering access to resources and equipment) *Accountability to others promoted motivation*
Motivation	Social support	*Sociocultural environments* (including supportive family and friends enhancing motivation)
Motivation	Feedback and monitoring	*Access to knowledgeable exercise professionals* *Self-monitoring techniques*
Motivation	Goal setting, action planning and coping planning	*Regular*,* dedicated physical activity time* *Preparing strategies in advance*

*Domain of the COM-B model [25], used to frame interview findings according to: capability, opportunity, motivation and behavior change

### Factors influencing supported physical activity maintenance in people with LTCs

**Fig. 2:**
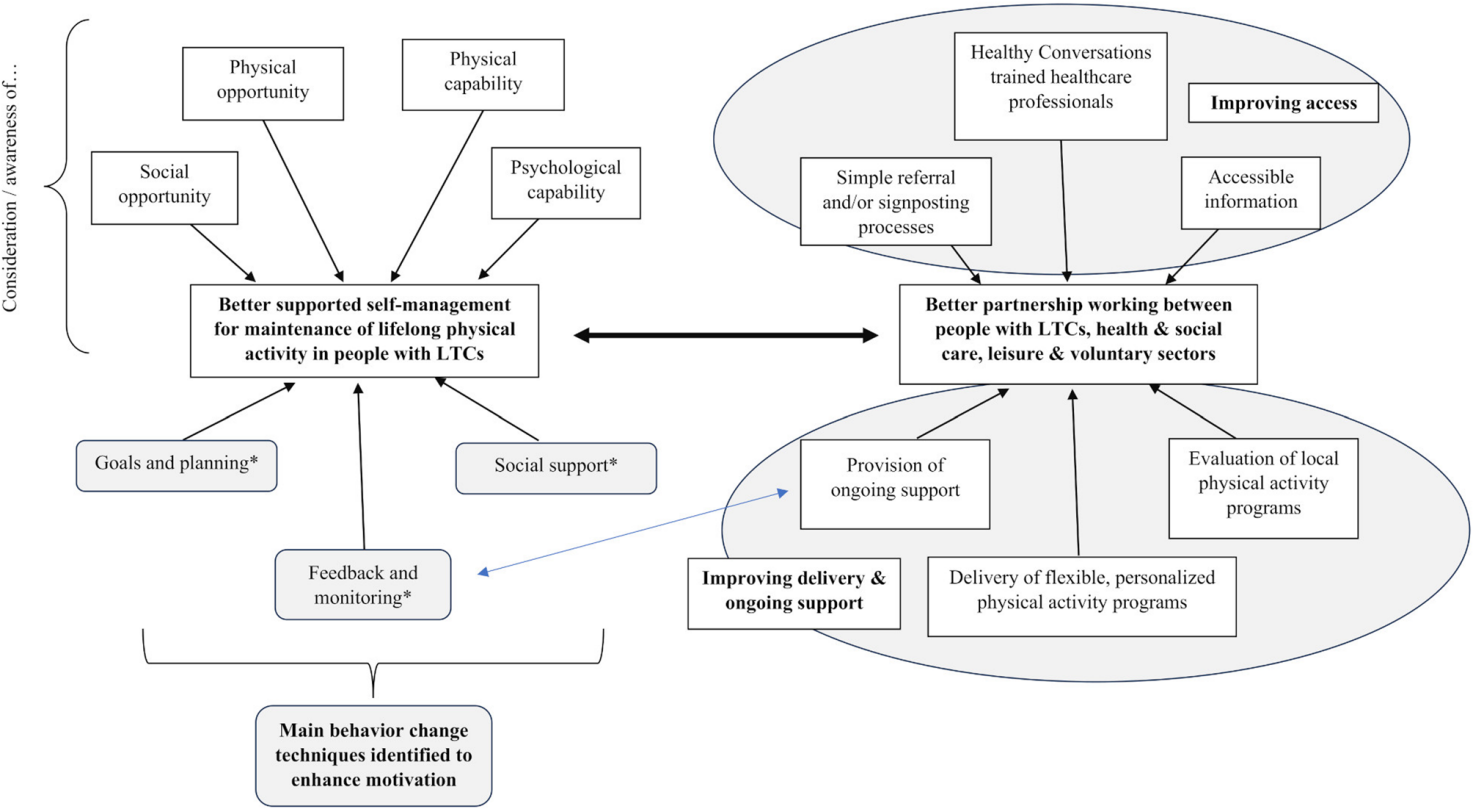
How can we best support people with long-term conditions (LTCs) to maintain physical activity? Schematic map of themes from interviews and workshops *Domain of the COM-B model [a^25^], used to frame interview findings. For behavior to occur, three components are required to take place: capability, opportunity, motivation

Figure 2 summaries the themes and sub-themes identified from the interviews and workshop, and the relationships between these. It suggests that to support physical activity maintenance for people with LTCs, there must be better partnership working between multi-sectoral agencies involved in the commissioning, design, referral and/or delivery of PARS, including post-PARS support in the community. It also emphasizes the importance of ensuring easy access to such schemes, and that the schemes support individuals to develop autonomy to become independently active.

### Physical and psychological capabilities

People with LTCs reported that the increased fitness and wellbeing that came from being active, enhanced their psychological capability to understand the importance of physical activity and physical capability to manage their conditions better (Table 2).

*“…I just feel so much better when I’ve exercised. I sleep better…I’ve got more energy; my appetite is better. Everything is better if I go…that’s my spur”.*

Woman (respiratory condition).

*“I definitely feel healthier. I’m quicker across the ground now when I’m walking whereas before it was a slow plod…I feel a lot better. I can keep up with the grandkids now when we’re walking along”.*

Man (cardiovascular condition).

However, ageing and LTC symptoms impacted on participant’s regular physical activities, particularly knowledge of how active they had previously been. This demotivated some but many were motivated by the fear of losing independence.

Participating in organized exercise programs like PARS, also allowed and motivated people with LTCs to stay physically active in safe and supportive environments, particularly during ill-health and relapse. The knowledge, scheduled time and environment offered by PARS, also raised their awareness of the importance and necessity of maintaining regular physical activity.

*“…I’m not a natural athlete*,* I never have been*,* but it’s always nice for someone to come over and say well done…the motivation is not there without the help and support which is what the [PARS] gave me”.*

Man (cardiovascular condition).

*“None of us like doing exercise*,* because exercise is not a thing we want to do at our age really … but we understand that it makes a huge difference to us”.*

Man (respiratory condition).

### Physical opportunities

Participants with LTCs also voiced some of the issues that restricted their opportunities to access PARS. There was a perception that the system limited access to one referral per year and once completed, some struggled without the support that the scheme offered them. All wanted support beyond its 12-month duration; they missed the feedback and monitoring they received from exercise professionals, which they felt made it difficult to maintain physical activity in the longer-term.

*“… it was costly to do the gymnasium [post PARS] … my wife and I were unable to meet that financial commitment both of us on my pension”.*

Man (musculoskeletal condition).

*“I did so well [at the gym]*,* after the three months I asked them if I could be referred again…once you’ve had one referral*,* they couldn’t do another one*,* until after a year*,* which did surprise me a bit … you didn’t get the support I thought you needed to motivate you to become a habit”.*

Man (cardiovascular condition).

Healthcare professionals felt that their opportunities to refer their patients and clients to PARS were limited by the time it took to search for services in the community, either free and paid for, and to navigate referral processes. Different organizations having different referral processes complicated this. They also felt that PARS were not equally accessible by, or advertised to, the general population.

*“But in all honesty*,* it’s just not well publicized what services are available for patients*,* to someone who is seeing them in clinic*,* or speaking to them on the phone*,* and then finding out ‘oh sorry your postcode doesn’t have…’*,* or ‘…it’s not running because of COVID’”.*

Man (GP, Hampshire).

*“…but really looking at that equity and access and realizing that people aren’t going to maintain if they can’t start…we’re adding to the inequality gap if we don’t change what we are doing”.*

Woman (leisure professional, Hampshire).

However, some healthcare professionals reported community and exercise providers to be overly cautious about including patients with LTCs in their PARS, because of their fear of worsening their conditions(s).

*“…we often have people coming from gyms saying they have told me to come about X and Y and we have no idea why they are worried…because in the vast majority of healthcare conditions exercises are only beneficial…I think as a society we medicalize things often that shouldn’t be medicalized. It makes our jobs harder and patients’ quality of life not as good”.*

Women (GP, Hampshire).

*“…for a lot of our referrals we don’t have a medical professional sign off. I think if they’re [requiring a] Level 4 [instructor]*,* that’s a different matter…it’s largely down to an insurance decision”.*

Man (leisure professional, Dorset and Hampshire).

Healthcare professionals felt this to be motivated by misunderstanding of the nature of LTCs, which had the effect of restricting physical activity choices for participants with LTCs, limiting their opportunities to improve their longer-term health and wellbeing.

### Social opportunities

PARS presented numerous social opportunities, including dedicated activity time, group sessions and post-session refreshments. These were time and opportunities for sharing experiences, knowledge, and entrenching habits for physical activity supported through relationships with other people with LTCs. Some PARS did not include opportunities to build exercise habits outside group sessions, however. A sense of belonging, enjoyment, commitment and accountability were valued by participants with LTCs and were factors conducive to maintaining activity in the longer term.

*“I think combining the physical exercise with the opportunity for the social interaction is a big improvement on just doing the exercise…It’s very useful to talk to other people who are meeting the same challenges or similar challenges because of course we are all different”.*

Man (neurological condition).

*“By having this weekly session*,* it puts some structure into the week…it’s less easy to dismiss and say oh I’m too busy this week…you make time for it”.*

Man (cardiovascular condition).

### Social support

However, healthcare professionals also described features of PARS that motivated them to refer; the group nature of the activities, the enjoyment of these, and the social support clients received from the group were crucial features in supporting physical activity maintenance.

*“…I think engagement*,* and I do think*,* if we are talking about exercise*,* I’m a great believer in group exercise and the social aspect”.*

Woman (GP, Hampshire).

Participants with LTCs also found that social support, in the form of feedback from knowledgeable exercise professionals, gave them confidence and reassurance to maintain their physical activity.

*“…if you are doing it on your own you don’t know whether you are being supportive or doing the right exercises but with the scheme…it actually is very supportive, and you don’t feel as if you are on your own…*null

Man (musculoskeletal condition).

*“It’s that reassurance you need because it’s such a major thing to go through…it’s very comforting to know if there is a slight problem you can just mention it and that’s fine”.*

Woman (cardiovascular condition).

### Feedback and monitoring

Continued support involving feedback and monitoring on physical activity maintenance, was raised by participants with LTCs and professionals as a priority in maintaining motivation and helping personalize care. This included both professional monitoring via in-person appointments, and via digital tools.

*“I was worried about being left on my own and not carrying on [after finishing pulmonary rehabilitation…I thought yes this is for me because I want someone to keep an eye on me.”*

Woman (respiratory condition).

*…it would need some sort of follow-up or contact with a patient to see how they were going, and to maybe re-motivate if they were falling off the wagon a bit…without a digital element to it that would be challenging to do…also some way of the patient relaying their progress to themselves or to you or both….*

Man (GP, Dorset).

Healthcare professionals also experienced a lack of communication and feedback about progress made by their patients from those running PARS, causing them to hesitate before making further referrals.

*“It’s a bit fiddly…and not particularly intuitive…I haven’t yet had any feedback from people I’ve referred. I’m interested to see how it ends up but I’ve kind of mixed feelings about how easy that system is…”*.

Man (GP, Hampshire).

A lack of feedback to healthcare professionals inhibited the patient-clinician relationship, and clinical conversations for ongoing support regarding goal setting and other behavior change techniques.

### Goal setting, action planning and coping planning

Ongoing support with goal setting and action planning was consistently seen by people with LTCs and multi-sectoral professionals as key to maintaining physical activity. This included coping planning, or ‘*If-Then planning’*, to help those with LTCs employ plans for situations like symptom flare-ups, that would allow them to maintain physical activities independently in their own communities.

*“…if you can’t do what you were planning to do*,* then do something else that may be less*,* or something that would affect you in a different way”.*

Man (PPI partner).

…so giving people permission to fail but not to stop…letting people know that failure is normal and that’s OK, but don’t give up and having some kind of support to go back to….

Woman (health and social care professional, Dorset).

However, few participants with LTCs adopted self-monitoring techniques; they were more reliant on informal conversations with their exercise and/or healthcare professionals to help them maintain activity.

*“…so it’s up to you to say well actually could I have a chat about [my progress]. Now I’m quite good about that but I’m not sure how much the others are. I suspect that some of the others don’t do very much of that”.*

Man (neurological condition).

### Factors contributing to an integrated partnership between healthcare, social and community professionals to support physical activity maintenance for people with LTCs

Based on the need identified in the interview analysis for better multi-sectoral partnership working, ease of access, and changing physical activity behavior (Fig. 2), there was consensus that the most effective single intervention to improve physical activity maintenance for LTCs would be to integrate care and support across the whole system. This was described as a pathway from medical intervention to community-based physical activity support. Subsequent workshop discussions focused on what factors would support better multi-sectoral partnership working to create such a pathway and are presented below.

### Simple referral or signposting process

LTC and professional stakeholders agreed that health and social care services were the starting point from which individuals were subsequently referred-on to leisure and community services, with healthcare available thereafter for condition-specific support. Predominantly, stakeholders felt that support for physical activity maintenance needed to be personalized and made appropriate to accommodate the LTC symptoms of the individual.

Part of the problem lay in poor inter-service communication, which participants with LTCs found particularly challenging when transitioning between health and community services. In some cases, this resulted in poorly coordinated care which was perceived to be impersonal (e.g., delayed and/or no follow-up). Participants with LTCs reported lack of consistency in the physical activity support they received as they transitioned between services. This lack of continuity and personalization acted to demotivate individuals, placed additional burdens on them, and limited their physical activity engagement.

*“Those are the things that really get to me. It’s the communication [between services] and I mean it’s just misinformation that people get fed up so much”.*

Woman (respiratory condition).

To optimize transitioning of participants between services, participants with LTCs and professionals spoke about the need for simple and clear processes for referral and signposting clients to appropriate services to support maintenance of their physical activity. This need was being responded to in some locations:

*“We are trying to set ourselves up as a [PARS] hub for Dorset. So it’s one form for the GPs and other health professionals to refer…”*

Woman (leisure professional).

*“We use ‘Refer All’…so health professionals can refer in through a secure link to us, providing the information on those individuals…”.*

Woman (leisure professional).

*“…lots of providers are using an open referral process and finding that’s really successful for them…they’ve looked at risk assessments in a different way and so are managing to get round that without needing a GP sign off and what we’re finding is that just reduces barriers for people to come and engage…”.*

Woman (voluntary/community professional, Hampshire).

### Accessible information

Alongside easily navigable referral and signposting processes, most multi-sectoral professionals emphasized the need for quick and clear access to information on various physical activity services within their communities. They discussed online directories and repositories that covered geographical areas and involved multiple services and sectors, allowing both LTC patients and professionals to identify appropriate, supported physical activities in the community. Existing directories were, however, perceived to be limited mainly by the practicalities of maintaining them.

*“I think the directory sounds like an obvious solution. I think in reality it just doesn’t work. I mean we’ve been looking at this for 20 years and every area is so different…there’s such variety and it also changes quite frequently…it would be virtually impossible to maintain”…*

Man (leisure professional, Dorset and Hampshire).

### Healthy conversations trained healthcare professionals

Primary healthcare professionals were seen as the most well-placed and equipped professionals to provide initial support for people with LTCs in their journeys to maintain physical activity, particularly in transition between services. Often the first point of contact for LTC patients to discuss long-term health and wellbeing, healthcare professionals recognized that the quality of the conversations they had were important in influencing how supported the patient felt, the physical activity services they accessed, and the benefits they gained. They emphasized the importance of managing supportive and skillful conversations about physical activity, preferences and readiness to engage.

*…that healthcare professionals are trained to understand how to identify someone’s readiness to engage but then also there’s… the flexibility of options available to people in terms of different pathways that will suit different people…*

Woman (health and social care professional/academic, Dorset).

In practice, participants with LTCs reported that the quality of these conversations in maintaining physical activity was dependent on the skills of the individual practitioner, and inconsistent across health care services, partly due to healthcare professional time constraints and workload.

*“…I think it is a matter of having somebody who is interested in you who you can relate to who you can discuss things with…”.*

Man (neurological condition).

*“The NHS doesn’t treat people anymore. It tries to identify conditions and put you on a pathway and as far as it’s concerned it’s a tick box exercise and hopefully*,* you’ll go on the right pathway*,* you’ll get the right help and then you’ll be discharged and that’s it and we can tick all the boxes. Nobody will sit down and actually treat you as a whole person”.*

Man (musculoskeletal condition).

### Delivery of flexible, personalized physical activity programs

Recognizing that people’s capabilities with LTCs fluctuated with age and different conditions, the need for services to be flexible in meeting changing physical activity needs over an individual’s lifespan was recognized by leisure professionals but ultimately limited by staffing and facilities availability.

*“…within our leisure centers, often we’re restricted on time available for certain classes because we have to stick to a timetable…but we’ve got to be a bit more open and flexible with that, but also being aware that we have constraints in terms of staff availability or space…”.*

Woman (leisure professional, Hampshire).

Several participants with LTCs spoke about the need for personalized programs and services to support their needs, if their motivation was to be sustained over the longer term. This included professionals offering people options for physical activities within their community, alongside structured support from professional services, and fluidity in transitioning LTC patients along their own pathway. One clinical academic involved in a regional health and wellbeing program recognized the importance of professionals, individualizing services to the LTC patient’s needs to maintain physical activity.

*“…one thing we talk an awful lot about in personalized care is that one size fits nobody*,* and that if you just have one pathway*,* one process*,* or limited possibilities*,* it’s not going to suit everybody and that we need to meet people where they are in their readiness to adopt physical activity”.*

Woman (health and social care professional/academic, Dorset and Hampshire).

They also emphasized the importance of conversational skills, involving behavior change techniques, to assess individual capabilities and support development of life-long habits.

### Provision of ongoing support

When talking about what kind of ‘personalized pathway’ participants with LTCs wanted, they asked for ongoing support for physical activity offered by a variety of co-operating services and organizations, selected to meet the individual’s needs and preferences. Numerous participants with LTCs suggested ongoing feedback and monitoring was important in helping maintain physical activity behavior but was often lacking. Participants felt that this was short-term thinking.

*“…I think in some respects to create less problems for the NHS further down the line*,* if they’d have intervened and said how are you getting on*,* is there anything else that you need to do to make sure that you are going in the right direction*,* that would have saved them a lot of time”.*

Man (musculoskeletal condition).

Several health, social and exercise care professionals also recognized that workload pressures limited services’ capacity for long-term support for this patient group. People with LTCs were clear, however, that feedback and monitoring on their physical activity progress, was essential for fostering accountability as a key determinant of physical activity maintenance.

*“…you’ve got someone who knows what you are about looking over you*,* keeping an eye on you. And you get to know a group of people who go regularly and I continue to do it. If it wasn’t there…I don’t think I would have the discipline to do that sort of exercise regularly once a week…I’ve got plenty enough other things to distract me but that sort of keeps me honest by having a commitment to go regularly and I see people that I’m familiar with and we catch up with each other”.*

Man (cardiovascular condition).

Feedback and monitoring also had indirect benefits alongside supporting accountability, particularly developing trusting relationships between patients, and healthcare and exercise professionals. Monitoring meant that people with LTCs felt supported and accountable to the professional, and their LTC peers, in turn motivating them to continue engaging in activity long-term.

*“The thing is you go to the doctor’s in the first place, and they refer you to the leisure center. They do what they have to do, and I find, and this is what disappoints me, they don’t follow up on what they suggest”.*

Man (musculoskeletal condition).

Furthermore, healthcare professionals also recognized the importance of providing ongoing support in monitoring and feedback to maintain physical activity, but felt hindered by limited resources, particularly post-PARS when individuals exit the service.

*“…[we need] some way of the service itself maybe contacting the patient proactively just to check up on how they are going, do they need any additional support, do they need to be referred back in because I think that’s the bit where patients may not reach out once they’ve completed the thing…also the other way having somewhere the patient can appoint a contact whether it’s telephone or messaging or some way of contacting the service itself…”*.

Man (GP, Buckinghamshire).

### Evaluation of local physical activity programs

The majority of professional stakeholders reported limited evaluation of physical activity services and outcome sharing across the region, citing limited resources, including insufficient staffing and lack of integration of services. One of the difficulties with receiving no feedback on patients’ experiences with PARS, was that there was no formal evaluation of PARS, nor of it’s benefits. LTC patients and professionals were not benefiting from evaluation of the service and hence there was no service improvement regime.

“…*we send out evaluation to our patients…12 weeks after they’ve completed the scheme*,* 6 months…and 12 months after they’ve completed the scheme…the challenge with that is getting people to respond*…”.

Woman (leisure professional, Dorset).

*“…"the conversation about evaluation is crucial to know whether it’s proving actually in real terms effective and helpful for those who are taking part”.*

Man (PPI partner).

Healthcare professionals further believed that evaluation was important because it would reduce resource wastage, optimize existing services, highlight ‘good practice’ examples, share knowledge between services, and help secure funding for future service development to support physical activity maintenance.

## Discussion

This study identified two priorities in increasing maintenance of physical activity for people living with LTCs, following engagement with a PARS. Firstly, ensuring that the content and delivery of PARS promotes appropriate, supported physical activity maintenance; and secondly, establishing better partnership working between services to develop an integrated care pathway to physical activity maintenance for people with LTCs. People with LTCs having undertaken a PARS, benefited from increased physical and psychological capabilities, access to opportunities to develop physical activity routines, with social support from peers and professionals, regular feedback and monitoring, and understanding and acceptance of their condition(s). Barriers preventing people with LTCs from accessing PARS were membership costs, restrictions to numbers of referrals, and the time and effort required to attend sessions. Professionals across sectors perceived they were limited in resources, time taken to navigate referral processes, and for some exercise professionals, anxiety about working with people who had serious, life-limiting illnesses.

### Intervention components

There were intervention components deemed necessary to integrate into PARS to maintain physical activity behavior change long-term. These include regular feedback and monitoring up to 12 months post-intervention, well-established action plans and peer support groups [17, 43]. This study indicated that some of these components were evident in UK PARS, such as having social support, goal setting and action planning, and short-term feedback and monitoring. However, components such as being theoretically-informed and having opportunity to build habits through practice of physical activity skills in daily life were absent [17]. Based on Mino et al.’s [22] review of PARS components, the schemes included in this study did little to restructure the physical environment, offer graded tasks, prompts and cues, or support to reduce negative emotions and emotional consequences of living with LTCs. This confirms the observation that many PARS are not optimally designed for physical activity maintenance [43]. Interestingly, the majority of participants with LTCs in this study developed coping (or ‘If-Then’) planning through PARS but were not supported in self-monitoring, nor becoming independently active outside of social/group settings. Previous work has suggested that digital tools can support delivery of these, but that such tools must be accessible to both healthcare professionals and LTC patients, have usability, acceptability, and offer LTC-adaptability [28].

The follow-on stakeholder workshops highlighted the importance to the effectiveness of PARS, to train professionals in the use of behavior change skills in their conversations with LTC patients. The 5As framework [44] is an approach for clinicians to ‘Ask’ about current behavior, ‘Advise’ on change, ‘Assess’ readiness to change, ‘Assist’ with goal setting, and ‘Arrange’ follow-up. Training healthcare professionals in Healthy Conversation Skills, which focus on personalized support, feedback and monitoring, was identified as a priority in this study, in building an integrated pathway to support physical activity maintenance, and crucial in supporting autonomy and building self-efficacy in those with LTCs [43]. These findings concur with current LTC research in cancer [19], obesity [20] and osteoarthritis [21]. Healthcare professionals are the first point of contact for LTC patients’ entering a PARS, and therefore, responsible for initiating Healthy Conversations about behavior change prior to referral. However, limited consultation time, high workload, and a traditional medical model approach may prevent early intervention [28, 45]. Changing culture from an outcome-focused, prescription’ model towards a biopsychosocial approach, whereby healthcare professionals discuss with their patients the importance of select behavior change techniques (e.g., feedback and monitoring, with task grading) prior to PARS entry, may be a longer-term target for future programs.

Accountability among LTC peers and professionals was a key motivating factor, alongside flexibility, which was deemed crucial by our participants in managing fluctuations in symptoms, ageing and/or comorbidities. Flexibility again, can be supported by professional conversations, in terms of individualizing physical activities to promote choice (autonomy), mastery (competence), and social opportunities (relatedness), to motivate behavior [23]. Our interviews highlighted that accountability was promoted by social support from peers, specifically by having someone to talk to in feeding-back and monitoring. The InterWalk trial [46] shows how accountability can support motivation over 12-months. A group interval walking program (three 1-hour sessions weekly), InterWalk involved type 2 diabetics, who were initially externally motivated by commitment and accountability, which commuted to internal motivations and a sense of autonomy, as their capabilities and self-efficacy developed [16]. Future interventions should consider how to involve an element of accountability in an individual’s relationship with physical activity throughout life. Sport England’s Physical Literacy Consensus Statement [47] now advises healthcare and community professionals to understand the importance of physical (the movement/activity), social (connectedness/relatedness), cognitive, and affective (feelings) factors in an individual’s relationship with physical activity [47, 48].

COM-B offers a framework that can be retrofitted to PARS to identify deficits in scheme content and delivery. The major deficit identified by participants in this study, however, is the lack of support for physical activity maintenance once the PARS has ended. InterWalk provides a good example of how to support independence [46], by tapering-off supervised activity, whilst teaching participants to self-monitor their activity/walking using smartphones and wearable devices. Social support, feedback and monitoring (e.g., pedometry) were considered by our LTC and professional interviewees to be important components of programs for physical activity maintenance. However, follow-on workshops revealed a lack of opportunities within PARS to develop autonomy and coping plans to maintain behavior. One difficulty encountered in applying COM-B in this context was the lack of evidence on how to best support physical activity maintenance post-PARs and other time-limited schemes. Few studies are designed to assess factors that support long-term maintenance of physical activity in LTCs [49]. We recently found only 35% of studies continue physical activity support post-intervention, with around a third identifying behavior change techniques [43]. One way of delivering this kind of long-term support would be to train PARS professionals in skills to support behavior change that can be used from the initial healthcare professional consultation, through to the point at which patients exit the programmes, taking with them a well-developed maintenance plan. This could be supported by brief intervention and/or connections to local community navigators, such as health and wellbeing coaches, trusted by healthcare professionals and trained in behavior change skills [27]. This pathway from hospital to long-term community support, would need commitment and close partnership working between exercise professionals, healthcare professionals and community organizations [11, 27, 33]. The structures to support this kind of partnership would also need to be in place.

### Optimizing partnership working by multi-sectoral professionals

Integrated, collaborative partnerships between multi-sectoral service providers were seen as a key strategy to help professionals individualize services for their LTC communities. LiveWell Dorset [50] is a good example of infrastructure that has been set up to facilitate a collaborative response to members of the community, who need this kind of support. It is a free health and wellbeing program in South-West England, within which healthcare professionals are central to supporting behavior change, both in-person and digitally, via a central online hub open to patients and community-based professionals. LiveWell Dorset’s [50] online platform, which facilitates inter-professional communication between local healthcare professionals and health coaches, appears to have been embedded into existing IT systems and professional networks. Previous work has found this to be crucial to enable engagement of those working in primary care [28]. Alongside offering LTC patients online support and physical activity monitoring as a broader public health approach [50], it also provides opportunity for inter-professional learning in delivering healthy conversations with LTC clients. The UK NHS Make Every Contact Count [51, 52] is a national training program for healthcare professionals in skills to enable such conversations, and to support use of behavior change skills in daily encounters with patients. This aligns with Public Health Scotland’s Physical Activity Referral Standards [53] for workforce development, which advocates for professionals being trained in health behavior change skills so that they can equip clients with the knowledge, confidence and tools to maintain their own health.

By sharing learning and training, services could also help improve signposting to existing, local opportunities for people with LTCs [52, 53]. For example, group-based sessions promoting autonomy in activity choices, peer support offering feedback and a sense of belonging, and opportunities for professionals to develop greater understanding of their client’s conditions and physical activity behaviors. Investment in program evaluation was requested by participants in this study. Services could consider using checklist auditing referral programs and being able to identify effective components in evaluation [22]. National standards are available in Scotland to guide reviews of existing services, and inform future program development in the community as opposed to in acute care [53]. It is currently unclear how many services adopt these standards.

### Strengths and limitations

This research was strengthened in methodological rigor by validating our interview findings, in our subsequent workshop discussions with LTC patient and professional stakeholders. This helped ensure our interpretations reflected the realities as participants experienced them, with the workshops complementing the interviews through additional perspectives across social care, voluntary and community sectors. In data analysis, the COM-B model [25] brought understanding of the key determinants of behavior, and potential mechanisms to promote behavior change for physical activity maintenance. Limitations of the study related to recruitment and participant selection. For example, our participants with LTCs were white British and mostly retired, and recruited through their PARS exercise professionals based on their understanding, availability, and their willingness to participate. This likely limited our insights and is unlikely to reflect the experiences of the wider population with LTCs. It was difficult to access potential interviewees having participated in schemes other than PARS, and those having dropped out following initial PARS referral. Interviewing these individuals would have enhanced our understanding of barriers preventing individuals with LTCs from completing PARS, and interventions in general.

## Conclusions

This research identified two ways to support people with LTCs having undertaken a PARS to maintain physical activity: (i) ensure that individuals are appropriately supported to maintain behavior lifelong, and (ii) promote partnership working between people with LTCs and multi-sectoral professionals. Healthcare, leisure, and community sectors should enhance integrated working, through programs like LiveWell Dorset, to provide local physical activity opportunities that include social support, feedback and monitoring and facilitate professional-public collaboration. Future research is necessary to complete the co-design of integrated pathways, that include professional training in healthy conversation skills, to promote long-term behavior change and maintain physical activity for LTC patients.

*Domain of the COM-B model [25], used to frame interview findings. For behavior to take place, three components are required: capability, opportunity, motivation.

## Electronic supplementary material

Below is the link to the electronic supplementary material.

Supplementary Material 1

Supplementary Material 2

Supplementary Material 3

## Data Availability

All data contained within the manuscript and the supporting qualitative transcripts and thematic analyses (including field notes) can be accessed upon request from the research team. Additional information and data are included within the published Supplementary Material.

## Abbreviations

ARC Wessex: Applied Research Collaboration Wessex
COM-B model: Capability, Opportunity, Motivation–Behavior model
COPD: Chronic obstructive pulmonary disease
GPs: General practitioners
LTC: Long-term health condition
LTCs: Long-term conditions
MOTH program: Maintenance Of physical acTivity beHavior program
NHS: National Health Service
NICE: National Institute for Health and Care Excellence
NIHR: National Institute for Health Research
PPI: Patient and public involvement
UK: United Kingdom
5A’s framework: Ask, advise, assess, assist, arrange
