# Supplementary material for: Maintaining physical activity in people with long-term conditions following engagement in physical activity referral schemes: barriers, enablers, and intervention strategies
Source: Int J Behav Nutr Phys Act. 2025 Jul 23;22:102. doi: 10.1186/s12966-025-01802-y (PMC12285069; doi:10.1186/s12966-025-01802-y)
Supplement: Supplementary file 1 — Supplementary Material 1 [file 12966_2025_1802_MOESM1_ESM.doc]

**Interview guide (people with LTCs)**

**Title of study: Physical activity referral schemes and health and wellbeing services – maintenance intervention**

**Introduction**

Interviewee consent – discuss interviewee’s consent to be interviewed:

- Check interviewee is happy to go ahead with the interview
- Go through consent form (emphasize confidentiality)
- Check interviewee is happy for interview to be audio-recorded
- Ask if they have any questions or concerns before proceeding
- Work through each consent question if participant has not provided written consent

Purpose of the interview – We are interested in hearing about your experiences of taking part in the physical activity referral scheme and how this has affected you in the longer term.

**Discussion topics**

1. **Physical activity referral scheme involvement**

- What did you think when you first heard about the [INSERT SCHEME/SERVICE]?
  - Prompt – why did you want to take part?
- Can you tell me more about what happened during the scheme/program?
  - Prompt – What physical activities did you do? Who else was involved? Where? When? How often? And for how long?
- Can you tell me about the parts that helped you feel as if it was doing you some good and those that you felt were unnecessary or didn’t help?
  - Prompt – things that helped/didn’t help
- At the end of the scheme/program, did you feel that you had been given enough support to continue to be more active on your own?
  - Prompt – did you feel ready?
  - Did you feel you had the necessary skills/abilities?

1. **Current physical activity behavior**

- Now that you’ve finished the program, are you as physically active as you would like to be? If so, how do you manage to stay active from day to day? If not, what needs to happen to make regular physical activity easier?
- Are you physically active with others? Do other people give you support?
  - Prompt – practical/encouragement, etc (from family/friends/HCPs/exercise professionals).

1. **Barriers and facilitators to long-term engagement in physical activity**

- For those who describe increased physical activity levels:
  - What was it about the scheme/program that helped you to make these changes?
  - Have there been any challenges to staying active?
  - Are there continuing challenges?
  - Do you feel the scheme/program is accessible to people from all cultures and backgrounds? Prompt - what are the barriers/how could this be improved?
- For those who describe no change/decline in physical activity levels:
  - What could have been done differently within the scheme/program to help you continue to be more physically active?
    - Prompts – what are the main things that prevent you from being more active?
  - Do you feel the scheme/program is accessible to people from all cultures and backgrounds?
    - Prompt - what are the barriers/how could this be improved?

1. **Personal experiences**

- If you were running the scheme/program, would you make any changes to make it more effective and better?
  - Prompt – what things could be added to the scheme/program to help you to be as active as possible within your everyday life?

1. **Anything to add?**

- Is there anything you would like to add to what we have talked about but not yet discussed?
  - Prompt – related to the scheme/program e.g. resources, training for professionals, referral into the program.

**Interview guide (healthcare professionals)**

**Title of study: Physical activity referral schemes and health and wellbeing services – maintenance intervention**

**Introduction** – discuss interviewee’s consent to be interviewed:

- Check interviewee is happy to go ahead with the interview.
- Go through consent form (emphasize confidentiality)
- Check interviewee is happy for interview to be audio-recorded.
- Ask if they have any questions or concerns before proceeding.
- Work through each consent question if the participant has not provided written consent.

Purpose of the interview – We are interested in hearing about your experiences of supporting, recommending and/or delivering a non-digital intervention (such as a physical activity referral scheme) for people living with long-term conditions (LTCs).

We also want to know what you would like to see in a future health intervention to help people with LTCs maintain physical activity in the long-term.

**Discussion topics**

1. **Experience of supporting, recommending or participating in non-digital, physical activity program**

- Have you used, or recommended a non-digital health intervention or supported the implementation of a non-digital intervention into practice?
- Prompt - for example of non-digital|(e.g., a physical activity referral scheme [PARS*]).

1. **Post-PARS experiences and physical activity behavior maintenance**

- Have you recommended many people to the different exercise classes that go on with the various conditions?
- Follow up – how have you found the response to those programs?
- Are they feasible and sustainable?

1. **Barriers and facilitators for a future intervention to support long-term engagement in physical activity (post-PARS)**

- How will the barriers to an intervention be different for non-digital compared to digital?
- What makes a successful non-digital health intervention for people with a LTC to engage in regular physical activity after completing PARS?
- Prompt - what components would need to be included in this?

1. ********The term ‘PARS’ was used during interviews with healthcare professionals, but not during interviews with people living with LTCs, whereby the researcher avoided acronyms and kept in lay-language (‘physical activity referral schemes’).*
