# Supplementary material for: Maintaining physical activity in people with long-term conditions following engagement in physical activity referral schemes: barriers, enablers, and intervention strategies
Source: Int J Behav Nutr Phys Act. 2025 Jul 23;22:102. doi: 10.1186/s12966-025-01802-y (PMC12285069; doi:10.1186/s12966-025-01802-y)
Supplement: Supplementary file 2 — Supplementary Material 2 [file 12966_2025_1802_MOESM2_ESM.docx]

Additional file 2. Phase iii workshop schedules.

|  | **Workshops 1 & 2** | **Workshops 3 & 4** | **Workshops 5 & 6** |
| --- | --- | --- | --- |
| **Schedule (workshop #)** | AM (1) - Multi-sectoral professionals (online)  PM (2) - People with LTCs (online) | AM (3) - Multi-sectoral professionals (online)  PM (4) - People with LTCs (in-person) | AM (5) - Multi-sectoral professionals (online)  PM (6) - People with LTCs (in-person) |
| **Aims** | - Introduce the purpose of the non-digital project within the MOTH program. - Learn about experiences and generate discussion around PARS. - Present preliminary findings from talking with people who have taken part in a PARS. - Present preliminary findings from reviewing the research literature. - Develop ideas to be added to PARS to support physical activity maintenance. - Develop a plan of how these might be implemented. | - Feedback on workshops 1 & 2. - Build consensus on how to create change, to best support people with LTCs to maintain physical activity. - Build consensus on how best to implement into practice. | - Feedback on workshops 3 & 4. - Discuss physical activity program components that may support physical activity maintenance. - Discuss how best to carry out an evaluation of services demonstrating different types of partnership working. |
| **Workshop questions** | *Questions for workshops 1 & 2 only.*  1. What are your thoughts about the range of people we spoke to who were taking part in a PARS?  2. How do you think PARSs support people to maintain their physical activity levels once they have left the scheme?  3. What are your thoughts on the most beneficial components of a physical activity program that help to maintain physical activity?  4. What are your views on the outcomes that are most important?  5. What factors might help or hinder putting these into practice?  ******See* ***bold-type questions*** *below for additional workshop-specific questions* | | |
| **Content / activities** | - Introductions - Summary of mapping process - Preliminary findings from interviews - Preliminary findings from literature review (43) – main cluster of BCTs with positive findings - ***Does this reflect their experiences?** - Potential intervention components & rationale | - ***What behaviour the intervention should focus on?** - ***How best to deliver the intervention?** - Discuss updated interview and literature review (43) findings - Potential intervention components & rationale - Focus on implementation plan - Provide examples of campaign materials (e.g. the Richmond group, UK Moving medicine). | - Feedback findings from workshops 3 & 4 - Changes to visual map - Plan to focus on evaluation of good practice - Intervention components - Focus on implementation plan |

AM, morning; LTC, long-term conditions; MOTH, Maintenance Of physical acTivity beHavior program; PARS, physical activity referral scheme; PM, afternoon.
