# Supplementary material for: Maintaining physical activity in people with long-term conditions following engagement in physical activity referral schemes: barriers, enablers, and intervention strategies
Source: Int J Behav Nutr Phys Act. 2025 Jul 23;22:102. doi: 10.1186/s12966-025-01802-y (PMC12285069; doi:10.1186/s12966-025-01802-y)
Supplement: Supplementary file 3 — Supplementary Material 3 [file 12966_2025_1802_MOESM3_ESM.docx]

Additional file 3. Coding of transcripts of interviews with individuals living with long-term conditions (LTCs) and healthcare professionals, showing how the domains of COM-B were used to organise the data.

| **Sample** | **Domain*** | **Code** |
| --- | --- | --- |
| People with LTCs | Capability - physical and psychological | PARS improved self-management ability |
|  | Capability - physical and psychological | Greater understanding and acceptance of their diagnosis |
|  | Opportunity / Motivation | PARS offered opportunities and motivation |
|  | Opportunity | Access to PARS |
|  | Opportunity - environmental | On-going membership costs with service providers, including time and travel to activities |
| Healthcare professionals | Opportunity - environmental | Lack of resources |
|  | Opportunity - environmental | Lack of time |
|  | Motivation / Behavioral | Group activities |
|  | Behavioral | ‘Over-medicalization’ |

*Domains of the COM-B model (25), used to frame interview findings according to capability, opportunity, motivation and behaviors.
